# Synthesis, structure, and antimicrobial activity of heterocyclic phenylsulfonyl- and 4-aminophenylsulfonyl-carboximidamides

**DOI:** 10.1007/s00706-012-0769-6

**Published:** 2012-05-15

**Authors:** Katarzyna Gobis, Henryk Foks, Katarzyna Wiśniewska, Maria Dąbrowska-Szponar, Ewa Augustynowicz-Kopeć, Agnieszka Napiórkowska, Artur Sikorski

**Affiliations:** 1 Department of Organic Chemistry, Medical University of Gdańsk, Gdańsk, Poland; 2 Department of Medicinal Microbiology, Chair of Microbiology, Medical University of Gdańsk, Gdańsk, Poland; 3 Department of Microbiology, Institute of Tuberculosis and Pulmonary Diseases, Warsaw, Poland; 4 Department of Physical Chemistry, University of Gdańsk, Gdańsk, Poland

**Keywords:** Sulfonamidine, Heterocycles, Synthesis, Crystal structure, Antimicrobial activity, Structure–activity relationship

## Abstract

**Abstract:**

A series of novel phenylsulfonyl- and 4-aminophenylsulfonyl-carboximidamides were synthesized by condensation of sulfonamides with heterocyclic methyl carbimidates obtained from heterocyclic carbonitriles and used ‘at its inception.’ The molecular structure of the obtained compounds is discussed. Compounds possessing heterocyclic systems with a nitrogen atom in the α position to the functional group showed a different single-crystal structure than expected. The synthesized derivatives were evaluated for antimicrobial activities: tuberculostatic, antibacterial, and antifungal.

**Graphical Abstract:**

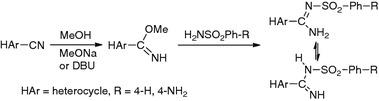
.

## Introduction

At the end of the twentieth century, a number of new and “reemerging” pathogens were recognized [1]. These included *S. pneumonia*, *L. pneumophila*, *M. avium*, *E. coli*, *H. pylori*, *S. aureus*, *C. albicans*, and *M. tuberculosis* [2–4]. These microorganisms quickly develop a multidrug resistance (MDR) to used chemotherapeutics and antibiotics. A special case is *M. tuberculosis*, whose strains also develop extensive drug-resistance (XDR). Resistant strains of microorganisms are a major threat to immunocompromised individuals, and infections caused by them are the most common complication in HIV-positive patients [5]. At the same time, a lack of development of new antimicrobial drugs is observed, which can pose a serious threat to public health [6]. Thus, the interest of many research groups is focused on the search for new drugs active against resistant strains.

One of the research directions is to modify the structure of already used drugs. So the interest in chemical groups such as, for example, sulfonamides has not diminished. This group is characterized by multidirectional pharmacological activity. Sulfonamides act as anhydrase inhibitors [7], antifungal [8], antiviral [9], anticancer [10], anti-inflammatory [11], and of course antibacterial agents.

Multidirectional biological activity also characterizes compounds possessing an amidine functional group. Amidine derivatives have anti-degenerative [12], antitumor [13], and anti-platelet effects [14]. Compounds with anti-HIV, antibacterial, and antifungal activities have also been found among them [15, 16].

There are few reports on the pharmacological activity of sulfonamidines. So far, only their in vitro ability to compete with triiodothyronine for binding to the thyroid hormone-α1 receptor (hTHR-α1) has been described [17]. These compounds can be obtained in several ways. They are formed as a result of the reaction of carbonitriles with primary sulfonamides [18] or in a reaction of amidines with sulfonyl chlorides [19]. The reports of reactions of sulfonamides with alkyl- or phenylcarbimidates could also be found in the chemical literature [20]. In the structure assigned to the products, two protons are connected to different nitrogen atoms of the amidine moiety [21]. That structure was adopted on the basis of ^1^H NMR spectra in which two different signals for those protons were observed. The reaction of sulfonamides with heterocyclic carbimidates has not been described so far.

The above facts prompt us to synthesize sulfonyl-carboximidamides possessing in their structure phenylsulfonyl or 4-aminophenylsulfonyl moieties linked to heterocyclic rings of pyridine, pyrimidine, or pyrazine by the sulfonamidine group. Synthesized compounds have been evaluated for their antimicrobial activity in vitro: tuberculostatic, antibacterial, and antifungal.

## Results and discussion

**Scheme 1:**
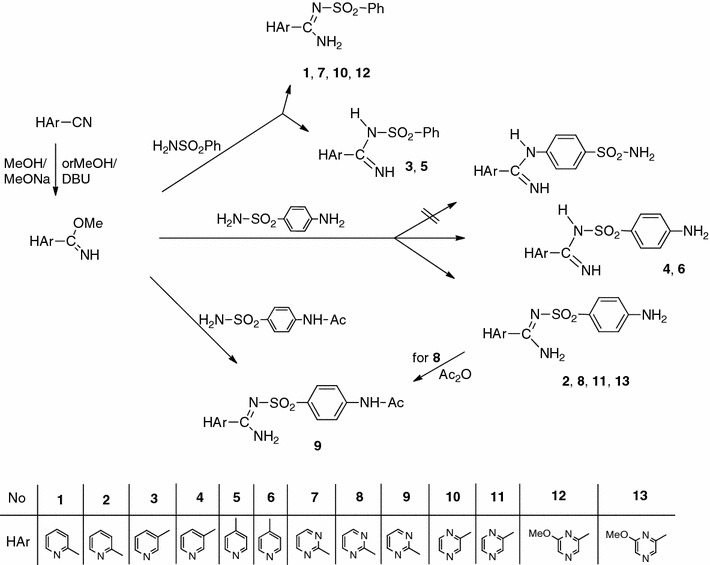

The subject of this work was the synthesis of heterocyclic phenylsulfonyl- and 4-aminophenylsulfonyl-carboximidamides **1**–**13**. The performed reactions are shown in Scheme 1.

The presented method of synthesis uses an intermediate such as carbimidate “at its inception,” and this is its main advantage. Carbimidates were obtained from the corresponding carbonitriles in methanol in the presence of 1,8-diazabicyclo[5.4.0]undec-7-ene (DBU) and without isolation underwent further reaction with benzene sulfonamide or 4-aminobenzenesulfonamide. The isolated carbimidates were used for the synthesis of pyrazine (**10**, **11**) and 6-methoxypyrazine (**12**, **13**) derivatives. They were obtained easily from 2-cyanopyrazine and 6-chloro-2-cyanopyrazine, respectively [22, 23]. Carbimidates were refluxed with benzene sulfonamide or 4-aminobenzenesulfonamide in diglyme (2-methoxyethyl ether) solution. All reactions proceeded with yields from moderate (38 %) to very good (83 %).

**Fig. 1:**
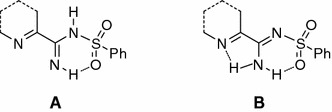
Possible systems of intramolecular hydrogen bonds in target molecules

The structures of all these new compounds were confirmed by IR and NMR spectra as well as elemental analyses. Two signals for the NH groups shifted from each other have been observed in the ^1^H NMR spectra. These separated signals can be due to the taken amino-imine structure of compounds obtained (Fig. 1, structure **a**) as we suggest for 3- and 4-pyridine derivatives **3**–**6**. They can also be a result of the magnetic inequivalence of NH protons in the amine moiety upon formation of a hydrogen bond in the case of the heterocyclic compounds in which the amidine group is in the α position to the nitrogen atom of heterocyclic ring (structure **b**). X-ray diffraction analysis was performed for *N*′-(4-aminophenylsulfonyl)-4-chloropicolinimidamide to address that question. We have described the synthesis of this compound previously [24]. It was chosen because we were able to obtain its crystals of sufficient size. The results of the single-crystal diffraction study confirmed a tautomeric structure **b** (Fig. 1). If both hydrogen atoms are bonded to the same nitrogen atom in the solid state, their magnetic inequivalence in the solution is probably caused by formation of hydrogen bonds and reduction of symmetry.

**Fig. 2:**
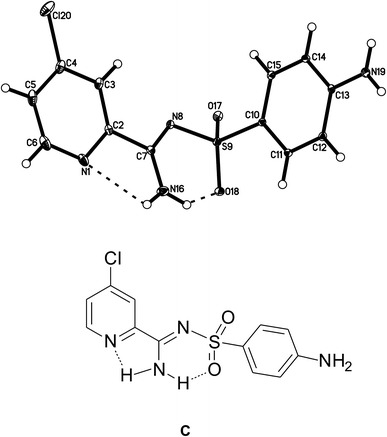
Structure of *N*′-(4-aminophenylsulfonyl)-4-chloropicolinimidamide showing 25 % probability displacements for ellipsoids. H atoms are shown as small spheres of arbitrary radius (intramolecular N–H···O and N–H···N interactions are represented by *dashed lines*)

The study also resolved the molecular structure of the products of the reaction between carbimidates and 4-aminobenzenesulfonamide, as the active group in that reaction could be both the amine group of the sulfonamide moiety, as it was in the case of the reaction of benzene sulfonamide, or the aromatic amine group in the *para* position to the sulfonamide moiety, since the reactions of aromatic and aliphatic amines with carbimidates have been described [25, 26]. For that purpose, the reaction of methyl pyrazine-2-carbimidate with *N*-(4-sulfamoylphenyl)acetamide was carried out. The resulting product **9** was identical with the compound that was obtained by the acetic anhydride acylation of derivative **8**, which was formed in the reaction of methyl pyrazine-2-carbimidate with 4-aminobenzenesulfonamide. This showed that the sulfonamide group was the active group in the reactions carried out, and the resulting compounds had structure **c** (Fig. 2).

### Crystal structure of *N*′-(4-aminophenylsulfonyl)-4-chloropicolinimidamide

The crystallographic data, data collection, and structure refinement of *N*′-(4-aminophenylsulfonyl)-4-chloropicolinimidamide are summarized in Table 1. The bond lengths and angles characterizing the geometry of the molecules are presented in Table 2.

**Table 1:** Crystal data and structure refinement for *N*′-(4-aminophenylsulfonyl)-4-chloropicolinimidamide

Empirical formula	C_12_H_11_ClN_4_O_2_S
Formula weight	310.76
Temperature/K	295 (2)
Wavelength/Å	Crystal system
Space group	0.71073
	Monoclinic
	*P*2_1_/c
Unit cell dimensions	
*a*/Å	14.6885 (7)
*b*/Å	5.7930 (3)
*c*/Å	16.0421 (9)
*β*/°	97.530 (5)
*V*/Å^3^	1353.25 (12)
*Z*	4
*D*_calc_/Mg m^−3^	1.525
Absorption coefficient/mm^−1^	0.443
*F*(000)	640
Crystal size/mm	0.35 × 0.15 × 0.10
Θ Range for data collection/°	3.08–25.00
Limiting indices	−14 ≤ *h* ≤ 17, −6 ≤ *k* ≤ 6, −19 ≤ *l* ≤ 13
Reflections collected/unique	8,544/2,373 (*R*_int_ = 0.0375)
Completeness 2*Θ* = 50.0/%	99.8
Refinement method	Full-matrix least-squares on *F* ^2^
Data/restraints/parameters	2,373/0/181
Goodness-of-fit on *F* ^2^	1.006
Final *R* indices [*I* > 2*σ*(*I*)]	*R*_1_ = 0.0406
	*wR*_2_ = 0.1013
*R* indices (all data)	*R*_1_ = 0.0626
	*wR*_2_ = 0.1060
Largest diff. peak and hole/e Å^−3^	0.369 and −0.345

**Table 2:** Selected bond lengths, valence angles, and torsion angles for *N*′-(4-aminophenylsulfonyl)-4-chloropicolinimidamide

Bond lengths/Å	
N1–C2	1.331(3)
N1–C6	1.338(5)
C2–C7	1.496(3)
C4–Cl20	1.724(4)
C5–C6	1.355(5)
C7–N16	1.308(3)
C7–N8	1.311(3)
N8–S9	1.626(2)
S9–C10	1.742(2)
C13–N19	1.369(3)
Valence angles/°	
C2–N1–C6	116.1(3)
N8–C7–C2	115.9(2)
C7–N8–S9	120.8(2)
N8–S9–C10	104.6(1)
Torsion angles/°	
N1–C2–C7–N8	164.6(2)
C3–C2–C7–N8	−16.0(4)
C2–C7–N8–S9	178.1(2)
C7–N8–S9–C10	−87.8(2)
N8–S9–C10–C11	108.4(2)

*N*′-(4-Aminophenylsulfonyl)-4-chloropicolinimidamide crystallized in the P2_1_/c monoclinic space group, with *a* = 14.6885(7) Å, *b* = 5.7930(3) Å, *c* = 16.0421(9) Å, and *β* = 97.530(5)°, *Z* = 4, and *V* = 1353.25(12) Å^3^.

In the molecule of the title compound (Fig. 2), the bond lengths and angles characterizing the geometry of the 4-aminophenylsulfonyl and pyridine fragments are typical for compounds containing them (Table 2).

**Fig. 3:**
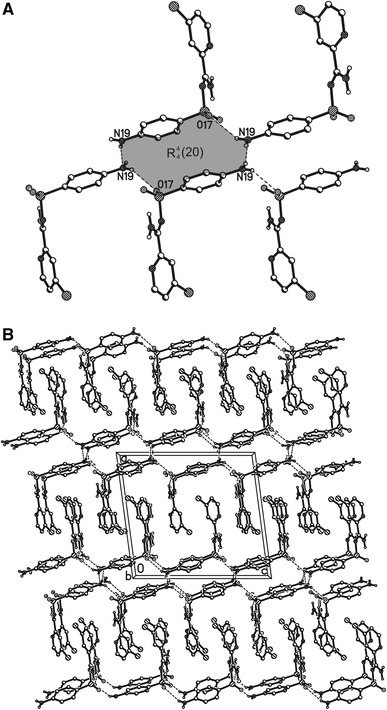
The $R_{4}^{4}$ (20) hydrogen bond motif (**a**) and the arrangement of the molecules in the crystal structure of *N*′-(4-aminophenylsulfonyl)-4-chloropicolinimidamide viewed along *b* axis (**b**). *Dashed lines*: N–H···O and N–H···N interactions. H atoms not involved in interactions omitted

In the crystal structure of *N*′-(4-aminophenylsulfonyl)-4-chloropicolinimidamide, the H atoms from the amino group bonded with the C7 atom participate in the intramolecular N16–H16A···O18 and N16–H16B···N1 hydrogen bond (Table 3; Fig. 2). In the packing, the molecules are linked into chains of rings along the *c* axis (Fig. 3b). In these rings, four molecules of *N*′-(4-aminophenylsulfonyl)-4-chloropicolinimidamide are linked via N19–H19A···O17 and N19–H19B···N19 and form the $R_{4}^{4}$ (20) hydrogen bond ring motif (Fig. 3a). The parallel lying chains of rings are connected through the N16–H16A···O17 hydrogen bond and form columns along the *b* axis (Table 3; Fig. 3a). In the crystal lattice, these columns form a zipper-type supramolecular motif.

**Table 3:** Hydrogen bonds for *N*′-(4-aminophenylsulfonyl)-4-chloropicolinimidamide with distances (d/Å): d(D···A) < R(D) + R(A) + 0.50 Å; d(H···A) < R(H) + R(A) − 0.12 Å and angle/° (<) < D–H···A > 100.0°

D–H	A	d(D–H)	d(H··A)	<D–H···A	d(D··A)
N16–H16A	O18^a^	0.86	2.15	2.758(3)	128
N16–H16B	N1^a^	0.86	2.26	2.630(3)	105
N16–H16A	O17^b^	0.86	2.37	2.988(3)	129
N19–H19A	O17^c^	0.86	2.31	3.035(3)	142
N19–H19B	N19^d^	0.86	2.58	3.424(3)	167

^a^Intramolecular H bond

Symmetry codes: ^b^ *x*, *y* − 1, *z*; ^c^ *x*, ½ −*y*, *z*− ½; ^d^ −*x*, *y*−½, ½ −*z*

### Tuberculostatic activity

The synthesized phenylsulfonyl- and 4-aminophenylsulfonyl-carboximidamides **1**–**13** were examined in vitro for their tuberculostatic activity against *M. tuberculosis* H_37_Rv strain and two “wild” strains isolated from tuberculosis patients: one (Spec. 210) resistant to *p*-aminosalicylic acid (PAS), isonicotinic acid hydrazide (INH), etambutol (ETB), and rifampicine (RFP), and the other (Spec. 192) fully sensitive to the administered tuberculostatics (Table 4).

**Table 4:** Antimicrobial activity of newly synthesized sulfonyl-carboximidamides **1**–**13**

No.	*MIC*/μg/cm^3^							
	*M. tuberculosis*			Bacterial strains			Fungal strains	
	H_37_Rv	192	210	*S. aureus*	*E. coli*	*P. aeruginosae*	*C. albicans*	*C. parapsilosis*
**1**	100	50	50	>256	>256	>256	>256	>256
**2**	100	50	50	>256	>256	>256	>256	>256
**3**	50	50	50	>256	>256	>256	>256	>256
**4**	50	50	50	>256	>256	>256	>256	>256
**5**	50	50	50	>256	>256	>256	>256	>256
**6**	50	50	50	>256	>256	>256	>256	>256
**7**	50	100	50	>256	>256	>256	>256	>256
**8**	100	100	50	>256	>256	>256	>256	>256
**9**	50	50	50	>256	>256	>256	>256	>256
**10**	100	50	50	>256	>256	>256	>256	>256
**11**	50	50	50	>256	>256	>256	>256	>256
**12**	50	50	50	>256	>256	>256	>256	>256
**13**	50	50	25	>256	>256	>256	>256	>256
INH	0.5	0.5	1.1	–	–	–	–	–

Minimum inhibitory concentrations for bacterial strains were determined by the two-fold serial dilution method for microdilution plates and for mycobacterial strains by two-fold classical test-tube method of successive dilution

*INH* isoniazid

Investigations were performed by a classical test-tube method of successive dilution in Youmans’ modification of Proskauer and Beck’s liquid medium containing 10 % of bovine serum [33, 34]. Bacterial suspensions were prepared from 14-day-old cultures of slowly growing strains and from 48-h-old cultures of saprophytic strains [35, 36]. Solutions of the compounds in ethylene glycol were tested. Stock solutions contained 10 mg of compounds in 1 cm^3^. Dilutions (in geometric progression) were prepared in Youmans’ medium. The medium containing no investigated substances and containing isoniazid (INH) as a reference drug were used for comparison. Incubation was performed at a temperature of 37 °C. The *MIC* values were determined as minimum concentration inhibiting the growth of tested tuberculous strains in relation to the probe with no tested compound. The influence of the compound on the growth of bacteria at a certain concentration, 3.1, 6.2, 12.5, 25, 50, and 100 μg/cm^3^, was evaluated.

The study showed that the newly synthesized sulfonylcarboximidamides **1**–**13** exhibited very low tuberculostatic activity. Minimumal inhibitory concentration (*MIC*) values for all the tested compounds ranged from 25 to 100 μg/cm^3^. No significant differences in compounds’ activity against the sensitive strain 192 and the resistant strain 210 have been observed. Isoniazid, the reference tuberculostatic, exhibited much higher activity with the *MIC* value 0.5–1.1 μg/cm^3^. These results classify the compounds tested as practically inactive against *M. tuberculosis*.

### Antibacterial and antifungal activities

Antibacterial and antifungal activities of newly synthesized compounds were also examined. In the study of antibacterial activity three recommended reference strains *S. aureus* ATCC 25923, *E. coli* ATCC 25922, and *P. aeruginosae* ATCC 27853 were used [37]. Antifungal activity was determined with use of two strains: *C. albicans* ATCC 90028 and *C. parapsilosis* ATCC 22019 [38]. The susceptibility of the microorganisms to the agents was determined by the broth microdilution assay according to the procedures outlined by the National Committee for Clinical Laboratory Standards [37, 38]. The stock solutions of the agents were prepared by dissolving the chemicals in DMSO. The final concentration of the agents in 200 mm^3^ of Mueller-Hinton broth (for bacterial strains) or in RPMI 1,640 (for fungi) ranged over 0.125–256 μg/cm^3^.

In order to prepare the bacterial suspension, overnight culture of bacteria in 3 % Triptic soy broth was diluted in sterile saline to the final concentration of approximately 10^7^ CFU/cm^3^. Aliquots (10 mm^3^) of bacterial suspension were added to each agent solution. The *MIC* was defined as the lowest concentration of the agent that completely inhibited growth of the bacteria after 18 h incubation at 35 °C.

Inocula of candida strains were prepared by suspension of five colonies picked from 24 h old cultures on Saburaud agar in sterile saline to the concentration of 10^6^ cells per cm^3^. The final concentration of the working suspension was approximately 10^4^ cells per cm^3^. Aliquots (10 mm^3^) of the suspension were added to each agar solution. The *MIC* was defined as the lowest concentration of the agent that completely inhibited growth of the fungi after 48 h incubation at 35 °C. The final results were average values from two independent experiments.

The study showed no antibacterial and antifungal activity of the tested compounds. All of the synthesized sulfonylcarboximidamides **1**–**13** exhibited activity with *MIC* > 256 μg/cm^3^, which meant that those values did not fit standard test concentrations.

## Conclusion

In conclusion, a series of novel sulfonyl-carboximidamides with different six-membered nitrogen heterocyclic systems were synthesized successfully in a reaction of heterocyclic methyl carbimidates with benzene sulfonamide and 4-aminobenzenesulfonamide. All these new compounds were confirmed by IR and NMR spectra as well as elemental analysis. The molecular structure of the obtained compounds was discussed. Compounds possessing heterocyclic systems with a nitrogen atom in the α position to the functional group showed a single-crystal structure different from expected and described for that chemical group in the literature. Antimicrobial activity of the synthesized compounds was evaluated against *M. tuberculosis*, *S. aureus*, *E. coli*, *P. aeruginosae*, *C. albicans*, and *C. parapsilosis*. Unfortunately, all of the studied compounds were practically inactive towards microbial strains tested.

## Experimental

All materials and solvents were of analytical reagent grade. Thin-layer chromatography was performed on Merck silica gel 60F_254_ plates and visualized with UV. The results of elemental analyses (C, H, N) for all obtained compounds were in agreement with calculated values within the range of ±0.3 %. ^1^H NMR spectra in CDCl_3_ or DMSO-*d*_6_ were recorded on Varian Unity Plus (500 MHz) and Varian Gemini (200 MHz) instruments (Varian, Palo Alto, CA). Infrared spectra were determined as KBr pellets of the solids on a Satellite FT-IR spectrophotometer (Mattson Instruments, Madison, WI). Melting points were determined with a Boethius apparatus (Franz Küstner Nachf. KG, Dresden, Germany). Methyl pyrazine-2-carbimidate and methyl 6-methoxypyrazine-2-carbimidate required for further syntheses were obtained according to the method described earlier by Foks and co-workers [22, 23].

### General method for the synthesis of sulfonyl-carboximidamides **1**–**8**

The respective carbonitrile (1 mmol) and 0.4 cm^3^ (2 mmol) of DBU were refluxed in 10 cm^3^ of methanol for 0.5 h. Then 0.8 mmol of benzene sulfonamide or 4-aminobenzenesulfonamide was added. The mixture was refluxed for another 3 h. Then methanol was evaporated in vacuo, and 30 cm^3^ of water was added to the residue. The precipitate of the product was filtered off, dried, and purified by recrystallization from a suitable solvent.

#### *N*′-*(Phenylsulfonyl)picolinimidamide* (**1**, C_12_H_11_N_3_O_2_S)

Recrystallization from ethanol afforded 138 mg (66 %) **1**. *M*.*p*.: 165–166 °C; *IR* (KBr): $\bar{\nu }$ = 3,432, 3,320 (ν N–H), 1,613, 1,538 (ν C = C), 1,280, 1,147 (ν SO_2_), 757 (γ C–H), 688 (γ N–H), 589, 557 cm^−1^; ^1^H NMR (200 MHz, CDCl_3_): δ = 7.43–7.59 (m, 4H, 3H Ph and 1H NH + D_2_O exchangeable), 7.82 (m, 1H, pyridine), 8.02 (m, 2H, Ph), 8.28 (m, 2H, pyridine), 8.33 (brs, 1H, NH + D_2_O exchangeable), 8.58 (m, 1H, pyridine) ppm; ^13^C NMR (50 MHz, CDCl_3_): *δ* = 123.10 (C-3), 126.38 (C-2′, C-6′), 127.82 (C-5), 129.32 (C-3′, C-5′), 132.71 (C-4′), 138.40 (C-4), 142.39 (C-1′), 148.71 (C-6), 149.20 (C-2), 159.20 (C = N) ppm.

#### *N*′-*(4*-*Aminophenylsulfonyl)picolinimidamide* (**2**, C_12_H_12_N_4_O_2_S)

Recrystallization from dioxane afforded 124 mg (56 %) **2**. *M.p*.: 202–205 °C; *IR* (KBr): $\bar{\nu }$ = 3,435, 3,400, 3,323, 3,253 (ν N–H), 1,610, 1,588 (ν C = C), 1,271, 1,144 (ν SO_2_), 1,091 (δ C–H), 821 (γ C–H), 566 (γ N–H) cm^−1^; ^1^H NMR (200 MHz, CDCl_3_): *δ* = 5.94 (s, 2H, NH_2_ + D_2_O exchangeable), 6.52 (d, 2H, Ph, *J* = 8.6 Hz), 7.57 (d, 2H, Ph, *J* = 8.8 Hz), 7.63–7.67 (m, 1H, pyridine), 7.93–8.11 (m, 3H, 2H pyridine and 1H NH + D_2_O exchangeable), 8.67 (d, 1H, pyridine, *J* = 4.8 Hz), 8.84 (brs, 1H, NH + D_2_O exchangeable) ppm; ^13^C NMR (50 MHz, DMSO-*d*_6_): *δ* = 112.79 (C-3′, C-5′), 122.83 (C-3), 127.57 (C-5), 128.38 (C-2′, C-6′), 138.26 (C-4, C-1′), 149.08 (C-6, C-4′), 152.93 (C-2), 158.04 (C = N) ppm.

#### *N*′-*(Phenylsulfonyl)nicotinimidamide* (**3**, C_12_H_11_N_3_O_2_S)

Recrystallization from dioxane–methanol mixture (1:1) afforded 98 mg (47 %) **3**. *M.p*.: 176–178 °C; *IR* (KBr): $\bar{\nu }$ = 3,439, 3,322 (ν N–H), 3,054 (ν C–H), 1,618, 1,518 (ν C = C), 1,274, 1,164, 1,149 (ν SO_2_), 825, 789 (γ C–H), 583 (γ N–H), 561 cm^−1^; ^1^H NMR (200 MHz, DMSO-*d*_6_): *δ* = 7.47–7.66 (m, 4H, Ph), 7.94–7.98 (m, 2H, Ph), 8.16–8.20 (m, 1H, pyridine), 8.30–8.60 (brs, 1H, NH + D_2_O exchangeable), 8.73–8.75 (m, 1H, pyridine), 8.98 (d, 1H, pyridine, *J* = 1.47 Hz), 9.10–9.40 (brs, 1H, NH + D_2_O exchangeable) ppm; ^13^C NMR (50 MHz, DMSO-*d*_6_): *δ* = 123.71 (C-4), 125.83 (C-2′, C-6′), 126.40 (C-3′, C-5′), 129.67 (C-3), 132.06 (C-4′), 132.56 (C-4), 135.99 (C-1′), 148.96 (C-2), 153.05 (C-6), 161.39 (C = N) ppm.

#### *N*′-*(4*-*Aminophenylsulfonyl)nicotinimidamide* (**4**, C_12_H_12_N_4_O_2_S)

Recrystallization from dioxane–ethanol mixture (1:1) afforded 132 mg (60 %) **4**. *M.p*.: 215–217 °C; *IR* (KBr): $\bar{\nu }$ = 3,448, 3,394, 3,337, 3,313, 3,248 (ν N–H), 2,923, 2,851 (ν C–H), 1,643, 1,612, 1,591, 1,528 (ν C = C), 1,269, 1,141 (ν SO_2_), 1,089 (δ C–H), 786, 698 (γ C–H), 562 (γ N–H) cm^−1^; ^1^H NMR (200 MHz, DMSO-*d*_6_): *δ* = 5.92 (s, 2H, NH_2_ + D_2_O exchangeable), 6.58 (d, 2H, Ph, *J* = 8.7 Hz), 7.46–7.52 (m, 1H, pyridine), 7.58 (d, 2H, Ph, *J* = 8.7 Hz), 8.12–8.18 (m, 2H, 1H pyridine and 1H NH + D_2_O exchangeable), 8.70–8.73 (m, 1H, pyridine), 8.95 (d, 1H, pyridine, *J* = 1.9 Hz), 9.02 (s, 1H, NH + D_2_O exchangeable) ppm; ^13^C NMR (50 MHz, DMSO-*d*_6_): *δ* = 112.76 (C-3′, C-5′), 123.68 (C-5), 127.81 (C-3), 128.39 (C-2′, C-6′), 129.86 (C-1′), 135.84 (C-2, C-4), 148.85 (C-6), 152.82 (C-4′), 160.13 (C = N) ppm.

#### *N*′-*(Phenylsulfonyl)isonicotinimidamide* (**5**, C_12_H_11_N_3_O_2_S)

Recrystallization from methanol–water mixture (1:1) afforded 111 mg (53%) **5**. *M.p*.: 155–156 °C; *IR* (KBr): $\bar{\nu }$ = 3,379 (ν N–H), 3,058, 2,925 (ν C–H), 1,644, 1,530 (ν C = C), 1,281, 1,142 (ν SO_2_), 1,086 (δ C–H), 843 (γ C–H), 589 (γ N–H), 556 cm^−1^; ^1^H NMR (200 MHz, DMSO-*d*_6_): *δ* = 7.54–7.76 (m, 5H, Ph), 7.96 (d, 2H, pyridine, *J* = 6.6 Hz), 8.49 (s, 1H, NH + D_2_O exchangeable), 8.71 (d, 2H, pyridine, *J* = 5 Hz), 9.30 (brs, 1H, NH + D_2_O exchangeable) ppm; ^13^C NMR (50 MHz, DMSO-*d*_6_): *δ* = 121.90 (C-3, C-5), 126.42 (C-2′, C-6′), 129.27 (C-3′, C-5′), 132.65 (C-4′), 141.26 (C-4), 142.41 (C-1′), 150.52 (C-2, C-6), 161.23 (C = N) ppm.

#### *N*′-*(4*-*Aminophenylsulfonyl)isonicotinimidamide* (**6**, C_12_H_12_N_4_O_2_S)

Recrystallization from dioxane–ethanol mixture (1:1) afforded 152 mg (69 %) **6**. *M.p*.: 226–229 °C; *IR* (KBr): $\bar{\nu }$ = 3,441, 3,357, 3,242 (ν N–H), 2,957, 2,849 (ν C–H), 1,644, 1,596, 1,527 (ν C = C), 1,276, 1,136 (ν SO_2_), 1,084 (δ C–H), 828 (γ C–H), 556 (γ N–H) cm^−1^; ^1^H NMR (500 MHz, DMSO-*d*_6_): *δ* = 5.96 (s, 2H, NH_2_ + D_2_O exchangeable), 6.59 (d, 2H, *J* = 8.8 Hz), 7.74 (d, 2H, pyridine, *J* = 5.9 Hz), 8.25 (brs, 1H, NH + D_2_O exchangeable), 8.71 (d, 2H, pyridine, *J* = 5.9 Hz), 9.10 (brs, 1H, NH + D_2_O exchangeable) ppm; ^13^C NMR (50 MHz, DMSO-*d*_6_): *δ* = 112.78 (C-3′, C-5′), 121.95 (C-3, C-5), 126.43 (C-2′, C-6′), 129.87 (C-1′), 141.36 (C-4), 150.48 (C-2, C-6), 152.85 (C-4′), 161.28 (C = N) ppm.

#### *N*′-*(Phenylsulfonyl)pyrimidine*-*2*-*carboximidamide* (**7**, C_11_H_10_N_4_O_2_S)

Recrystallization from dioxane afforded 107 mg (51 %) **7**. *M.p*.: 206–208 °C; *IR* (KBr): $\bar{\nu }$ = 3,396, 3,330 (ν N–H), 1,621,1,554 (ν C = C), 1,280, 1,151 (ν SO_2_), 833, 790, 689 (γ C–H), 590 (γ N–H), 501 cm^−1^; ^1^H NMR (200 MHz, DMSO-*d*_6_): *δ* = 7.54–7.73 (4H, 3H Ph and 1H pyrimidine), 7.91 (d, 2H, Ph, *J* = 8.2 Hz), 8.21 (brs, 1H, NH + D_2_O exchangeable), 8.53 (brs, 1H, NH + D_2_O exchangeable), 8.96 (d, 2H, pyrimidine, *J* = 4.6 Hz) ppm; ^13^C NMR (50 MHz, DMSO-*d*_6_): *δ* = 123.58 (C-5), 126.50 (C-2′, C-6′), 129.27 (C-3′, C-5′), 132.68 (C-4′), 142.39 (C-1′), 158.11 (C-4, C-6), 158.53 (C-2), 159.24 (C = N) ppm.

#### *N*′-*(4*-*Aminophenylsulfonyl)pyrimidine*-*2*-*carboximidamide* (**8**, C_11_H_11_N_5_O_2_S)

Recrystallization from ethylene glycol–methanol mixture (1:1) afforded 175 mg (79 %) **8**. *M.p*.: 259–261 °C; *IR* (KBr): $\bar{\nu }$ = 3,380, 3,330, 3,237 (ν N–H), 1,621, 1,592, 1,562, 1,503, 1,391 (ν C = C), 1,268, 1,142 (ν SO_2_), 830, 787 (γ C–H), 678, 578 (γ N–H), 546 cm^−1^; ^1^H NMR (500 MHz, DMSO-*d*_6_): *δ* = 5.95 (s, 2H, NH_2_ + D_2_O exchangeable), 6.52 (d, 2H, Ph, *J* = 8.8 Hz), 7.54 (d, 2H, Ph, *J* = 8.8 Hz), 7.57 (t, 1H, pyrimidine, *J* = 4.8 Hz), 8.20 (brs, 1H, NH + D_2_O exchangeable), 8.87 (brs, 1H, NH + D_2_O exchangeable), 8.94 (d, 2H, pyrimidine, *J* = 4.8 Hz) ppm; ^13^C NMR (50 MHz, DMSO-*d*_6_): *δ* = 112.76 (C-3′, C-5′), 123.50 (C-5), 127.51 (C-1′), 128.54 (C-2′, C-6′), 152.96 (C-4′), 157.71 (C-2), 158.09 (C-4, C-6), 158.50 (C = N) ppm.

#### *N*′-*[4*-*[N*-*[Amino(pyrimidin*-*2*-*yl)methylene]sulfamoyl]phenyl]acetamide* (**9**, C_13_H_13_N_5_O_3_S)

Method A: the title compound was obtained according to the method described above for compounds **1**–**8** from 0.11 cm^3^ (1 mmol) of 2-cyanopyrimidine and 0.43 g (2 mmol) of *N*-(4-sulfamoylphenyl)acetamide affording 112 mg (35 %) **9**.

Method B: sulfonylcarboximidamide **8** (0.28 g, 1 mmol) was refluxed for 0.5 h in a solution of 0.5 cm^3^ (5 mmol) of acetic anhydride in 5 cm^3^ of pyridine. Then pyridine was evaporated in vacuo, and 20 g of ice was added to the residue. The precipitate was filtered off, dried, and recrystallized from ethylene glycol to afford 268 mg (84 %) **9**.

*M.p*.: 253–254 °C; *IR* (KBr): $\bar{\nu }$ = 3,385, 3,301 (ν N–H), 2,924, 2,854 (ν C–H), 1,684 (ν C = O), 1,624, 1,590, 1,562, 1,525, 1,401 (ν C = C), 1,280, 1,148 (ν SO_2_), 736 (γ C–H), 565 (γ N–H) cm^−1^; ^1^H NMR (500 MHz, DMSO-*d*_6_): *δ* = 2.09 (s, 3H, CH_3_), 7.72 (t, 1H, pyrimidine, *J* = 4.8 Hz), 7.75 (d, 2H, Ph, *J* = 8.8 Hz), 7.86 (d, 2H, Ph, *J* = 8.8 Hz), 8.42 (brs, 1H, NH + D_2_O exchangeable), 8.96 (d, 2H, pyrimidine, *J* = 4.1 Hz), 9.08 (brs, 1H, NH + D_2_O exchangeable), 10.33 (s, 1H, NH + D_2_O exchangeable) ppm; ^13^C NMR (50 MHz, DMSO-*d*_6_): *δ* = 24.39 (CH_3_), 118.73 (C-3′, C-5′), 123.58 (C-5), 127.73 (C-2′), 136.03 (C-1′), 143.08 (C-4′), 158.11 (C-4, C-6), 158.46 (C-2), 158.80 (C = N) ppm.

### General procedure for the synthesis of sulfonylcarboximidamides **10**–**13**

Methyl pyrazine-2-carbimidate or methyl 6-methoxypyrazine-2-carbimidate (3 mmol) and the respective sulfonamide (2.5 mmol) were refluxed in 5 cm^3^ of diglyme for 15 min. After cooling down 20 g of ice was added to the mixture, and the precipitate of the product was filtered off, dried, and purified by recrystallization from a suitable solvent with activated carbon.

#### *N*′-*(Phenylsulfonyl)pyrazine*-*2*-*carboximidamide* (**10**, C_11_H_10_N_4_O_2_S)

Recrystallization from dioxane afforded 249 mg (38 %) **10**. *M.p*.: 218–219 °C; *IR* (KBr): $\bar{\nu }$ = 3,434, 3,321 (ν N–H), 1,612, 1,545 (ν C = C), 1,278, 1,151 (ν SO_2_), 801, 686 (γ C–H), 590 (γ N–H) cm^−1^; ^1^H NMR (200 MHz, DMSO-*d*_6_): *δ* = 7.54–7.69 (m, 3H, Ph), 7.98 (d, 2H, Ph, *J* = 7.3 Hz), 8.44 (brs, 1H, NH + D_2_O exchangeable), 8.77 (s, 1H, pyrazine), 8.90 (s, 1H, pyrazine), 9.15 (brs, 1H, NH + D_2_O exchangeable), 9.23 (s, 1H, pyrazine) ppm; ^13^C NMR (50 MHz, DMSO-*d*_6_): *δ* = 126.49 (C-2′, C-6′), 129.35 (C-3′, C-5′), 132.84 (C-4′), 143.92 (C-2), 144.21 (C-3, C-5), 144.54 (C-1′), 148.49 (C-6), 158.28 (C = N) ppm.

#### *N*′-*(4*-*Aminophenylsulfonyl)pyrazine*-*2*-*carboximidamide* (**11**, C_11_H_11_N_5_O_2_S)

Recrystallization from dioxane afforded 381 mg (55 %) **11**. *M.p*.: 247–249 °C; *IR* (KBr): $\bar{\nu }$ = 3,431, 3,394, 3,320, 3,252 (ν N–H), 1,612, 1,593 (ν C = C), 1,268, 1,145 (ν SO_2_), 1,092 (δ C–H), 798 (γ C–H), 567 (γ N–H) cm^−1^; ^1^H NMR (200 MHz, DMSO-*d*_6_): *δ* = 5.97 (s, 2H, NH_2_ + D_2_O exchangeable), 6.57 (d, 2H, Ph, *J* = 8.7 Hz), 7.59 (d, 2H, Ph, *J* = 8.7 Hz), 8.14 (brs, 1H, NH + D_2_O exchangeable), 8.75 (d, 1H, pyrazine, *J* = 2.4 Hz), 8.89 (d, 1H, pyrazine, *J* = 2.4 Hz), 8.92 (brs, 1H, NH + D_2_O exchangeable), 9.20 (s, 1H, pyrazine) ppm; ^13^C NMR (50 MHz, DMSO-*d*_6_): *δ* = 112.82 (C-3′, C-5′), 128.53 (C-2′, C-6′), 143.83 (C-2), 144.02 (C-3, C-5), 144.78 (C-1′), 148.23 (C-6), 153.05 (C-4′), 157.05 (C = N) ppm.

#### *N*′-*(Phenylsulfonyl)*-*6*-*methoxypyrazine*-*2*-*carboximidamide* (**12**, C_12_H_12_N_4_O_3_S)

Recrystallization from ethanol afforded 584 mg (80 %) **12**. *M.p*.: 156–157 °C; *IR* (KBr): $\bar{\nu }$ = 3,395, 3,300 (ν N–H), 1,640, 1,580, 1,543 (ν C = C), 1,383 (δ C–H), 1,306, 1,144 (ν SO_2_), 1,008 (δ C–H), 803 (γ C–H), 591 (γ N–H) cm^−1^; ^1^H NMR (200 MHz, DMSO-*d*_6_): *δ* = 4.03 (s, 3H, OCH_3_), 7.53–7.65 (m, 3H, Ph), 8.00 (d, 2H, Ph, *J* = 7.6 Hz), 8.50 (brs, 1H, NH + D_2_O exchangeable), 8.53 (s, 1H, pyrazine), 8.77 (s, 1H, pyrazine), 9.02 (brs, 1H, NH + D_2_O exchangeable) ppm; ^13^C NMR (50 MHz, DMSO-*d*_6_): *δ* = 54.54 (OCH_3_), 126.44 (C-2′, C-6′), 129.33 (C-3′, C-5′), 132.77 (C-3), 135.61 (C-4′), 139.67 (C-2), 140.88 (C-1′), 142.24 (C-5), 158.27 (C-6), 159.01 (C = N) ppm.

#### *N*′-*(4*-*Aminophenylsulfonyl)*-*6*-*methoxypyrazine*-*2*-*carboximidamide* (**13**, C_12_H_13_N_5_O_3_S)

Recrystallization from methanol afforded 637 mg (83 %) **13**. *M.p*.: 188–189 °C; *IR* (KBr): $\bar{\nu }$ = 3,468, 3,417, 3,370, 3,309, 3,244 (ν N–H), 1,634, 1,584, 1,545 (ν C = C), 1,379 (δ C–H), 1,318, 1,261, 1,133 (ν SO_2_), 1,079 (δ C–H), 788 (γ C–H), 544 (γ N–H) cm^−1^; ^1^H NMR (200 MHz, DMSO-*d*_6_): *δ* = 4.02 (s, 3H, OCH_3_), 5.95 (s, 2H, NH_2_ + D_2_O exchangeable), 6.59 (d, 2H, Ph, *J* = 8.8 Hz), 7.59 (d, 2H, Ph, *J* = 8.5 Hz), 8.21 (s, 1H, NH + D_2_O exchangeable), 8.51 (s, 1H, pyrazine), 8.73 (s, 1H, pyrazine), 8.77 (s, 1H, NH + D_2_O exchangeable) ppm; ^13^C NMR (50 MHz, DMSO-*d*_6_): *δ* = 54.52 (OCH_3_), 112.76 (C-3′, C-5′), 128.56 (C-2′, C-6′), 132.74 (C-3), 139.84 (C-2), 141.76 (C-1′), 142.27 (C-5), 158.29 (C-6), 157.88 (C = N) ppm.

### Crystal structure of *N*′-(4-aminophenylsulfonyl)-4-chloropicolinimidamide

Single crystals of *N*′-(4-aminophenylsulfonyl)-4-chloropicolinimidamide suitable for X-ray diffraction were obtained from ethanol by slow evaporation of the solvent at room temperature. Good quality single-crystal specimens were selected for experiments at *T* = 295(2) K. They were mounted with epoxy glue at the tip of glass capillaries. Diffraction data were collected on an Oxford Diffraction Gemini R ULTRA Ruby CCD diffractometer with MoKα radiation (*λ* = 0.71073 Å). The lattice parameters were obtained by least-squares fit to the optimized setting angles of the collected reflections by means of CrysAlis CCD [27]. Data were reduced by using CrysAlis RED [27] software with applying multi-scan absorption corrections (empirical absorption correction using spherical harmonics, implemented in SCALE3 ABSPACK scaling algorithm). The structural resolution procedure was made using the SHELXS-97 package solving the structures by direct methods and carrying out refinements by full-matrix least-squares on *F* ^2^ using the SHELXL-97 program [28]. All H atoms bound with aromatic C atoms were placed geometrically and refined using a riding model with C–H = 0.93 Å and *U*_iso_(H) = 1.2 *U*_eq_(C). All H atoms bound with N atoms were placed geometrically and refined using a riding model with N–H = 0.86 Å and *U*_iso_(H) = 1.5 *U*_eq_(N). The –NH_2_ group containing the N19 atom was assumed to be planar-trigonal and coplanar with the mean plane of the benzene ring. The –NH_2_ group containing the N16 atom was assumed to be planar-trigonal and coplanar with the mean plane delineated by C2, C7, and N8 atoms. All interactions demonstrated were found by the PLATON program [29]. The programs used to prepare molecular graphics were: ORTEPII [30], PLUTO-78 [31], and Mercury [32]. Full crystallographic details, excluding structural features, have been deposited (deposition no. 849210) with the Cambridge Crystallographic Data Center. These data may be obtained, on request, from the Director, CCDC, 12 Union Road, Cambridge, CB2 1EZ, UK (Tel.: +44-1223-336408; Fax: +44-1223-336033; e-mail:deposit@ccdc.cam.ac.uk or http://www.ccdc.cam.ac.uk).
